# High-Frequency Synchronization Improves Firing Rate Contrast and Information Transmission Efficiency in E/I Neuronal Networks

**DOI:** 10.1155/2020/8823111

**Published:** 2020-11-09

**Authors:** Fang Han, Zhijie Wang, Hong Fan, Yaopeng Zhang

**Affiliations:** ^1^College of Information Science and Technology, Donghua University, Shanghai 201620, China; ^2^Glorious Sun School of Business and Management, Donghua University, Shanghai 200051, China; ^3^State Key Laboratory for Modification of Chemical Fibers and Polymer Materials, Shanghai Belt and Road Joint Laboratory of Advanced Fiber and Low-Dimension Materials, College of Materials Science and Engineering, Donghua University, Shanghai 201620, China

## Abstract

High-frequency synchronization has been found in many real neural systems and is confirmed by excitatory/inhibitory (E/I) network models. However, the functional role played by it remains elusive. In this paper, it is found that high-frequency synchronization in E/I neuronal networks could improve the firing rate contrast of the whole network, no matter if the network is fully connected or randomly connected, with noise or without noise. It is also found that the global firing rate contrast enhancement can prevent the number of spikes of the neurons measured within the limited time window from being confused by noise, thereby enhancing the information encoding efficiency (quantified by entropy theory here) of the neuronal system. The mechanism of firing rate contrast enhancement is also investigated. Our work implies a possible functional role in information transmission of high-frequency synchronization in neuronal systems.

## 1. Introduction

High-frequency synchronization of neural firing is believed to be one possible origination of high-frequency brain oscillations and gamma oscillations, which reside in the frequency band between 30 and 90 hertz and are found in many regions of the cerebral cortex [1]. Synchronization of neural firing may be relevant to some disorders of neural systems [2], but more importantly, the high-frequency oscillations (synchronization) are found to be related with many cognitive behaviors such as the processing of sensory signals, learning and memory, and attention in many biological experiments [3–7]. To reveal the underlying mechanisms of functional roles of the high-frequency synchronization (oscillations) on these cognitive behaviors, neural network models are constructed. Some functional roles of the high-frequency oscillations are hypothesized and tested with neural network models. One of the possible functional roles of the high-frequency oscillations is the feature binding [8–10]. Various features of the object such as shape, color, and motion are represented by different groups of neurons in different regions of the cerebral cortex. These groups of neurons fire synchronously, thereby distinguishing them from other neurons in the cerebral cortex. Thus, the corresponding features encoded in these synchronized firing of neurons are bound together to form a complete perceptual construct. Another possible function of the high-frequency oscillation is stimulus selection [11–14]. More coherent stimuli oscillating at high frequency are found to have a competitive advantage over less coherent ones; thus, the coherent stimuli are selected to pass down the neural systems.

Though these proposed functions of the high-frequency oscillation (synchronization) have been tested by neural network models, they are not producing satisfactory results due to the lack of relevant data and the oversimplicity of these models. Since neural systems are usually composed of both excitatory and inhibitory neurons, it is helpful to have a closer look at the mechanism of the genesis of high-frequency synchronization in E/I networks before we explore its functional role. It is widely believed that ING (interneuron gamma) and PING (pyramidal and interneuron gamma) are two possible mechanisms of the generation of high-frequency synchronization [15]. ING mechanism [16–19] suggests that interneurons (inhibitory neurons) themselves can induce synchronization, and this synchronization of inhibitory neurons makes excitatory neurons fire synchronously through the inhibitory synapses on excitatory neurons. However, as excitatory neurons have excitatory synapses on inhibitory neurons as well, excitatory neurons also affect the firing of the inhibitory neurons. For example, excitatory neurons with sensory input encoded in them may drive inhibitory neurons to fire more frequently through excitatory synapses [20]. The PING mechanism [21] supposes that inhibitory neurons themselves cannot generate high-frequency synchronization. It is the interaction of excitatory drive and inhibition that induces the synchronization. Furthermore, it is believed that only a fraction of neurons fires at each cycle of the synchronization [22, 23]. Therefore, a fraction of excitatory neurons fires at each cycle of the synchronization, which provides synaptic current helping to drive inhibitory neurons to fire. The firing of inhibitory neurons inhibits the firing of all neurons in the network. This reminds us of the lateral inhibition [24–26]. Lateral inhibition suggests that the firing of excitatory neurons drives the firing of the neighboring inhibitory neurons, which inhibits the firing of the neighboring excitatory neurons in turn. Inspired by this idea, in Ref. [27], we found that inhibitory neurons could act as a global recurrent inhibition to enhance the firing rate contrast of the whole neuronal network. This further reminds us that improving firing rate contrast could be a possible function of neural synchronization, and as information may be encoded in the firing rate of neurons [28], neural synchronization could contribute to information transmission of neural systems.

We proposed and confirmed this global contrast enhancement mechanism based on the high-frequency synchronization in the E/I network in this paper. More importantly, we confirmed by entropy theory [29–31] that the firing rate contrast enhancement can prevent the numbers of firings of the neurons measured within a limited time window from being confused by noise, thereby enhancing the information encoding efficiency of neural systems. This paper is organized as follows. Model and Methods provides the E/I network model and the methods for quantifying the neuronal information transmission efficiency. Simulation Results presents simulation and quantitative results on the enhancement of the firing rate contrast with relatively simple E/I networks. Mechanism of the Global Contrast Enhancement gives a qualitative analysis of the mechanism of the enhancement of firing rate contrast in E/I networks. Conclusions and Discussions summarizes the results and gives some discussions.

## 2. Model and Methods

**Figure 1:**
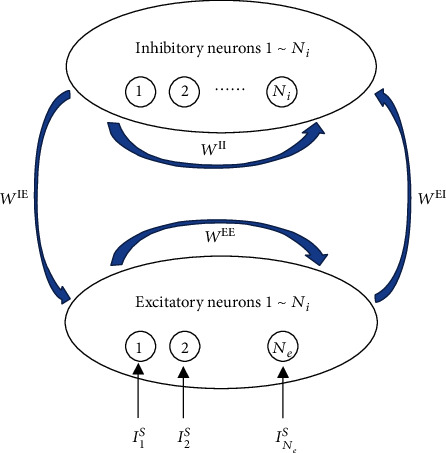
The structure of the E/I network.

The information encoded in the sensory neurons passes through the neural systems layer by layer. It is expected that information can be transmitted through the noisy neural systems with less information loss and less energy consumption. We proposed a method that is based on the entropy theory to quantify the neuronal information transmission efficiency. Suppose there is a group of neurons with population size *N*_*e*_ (see the excitatory neurons in Figure 1). Each neuron, for example, neuron *i*, receives an input *I*_*i*_^*S*^, the firing rate of which, *r*_*i*_, is measured by $\underset{T\to \infty }{\lim}k_{i}/T$ where *k*_*i*_ is the number of spikes emitted by the neuron during period *T*. When *T* is finite, *r*_*i*_ is usually a random number due to the uncertainty of the internal and external environment of the neuron. We hope that for any *I*_*i*_^S^ ≠ *I*_*j*_^S^, *r*_*i*_ ≠ *r*_*j*_, i.e., for any *I*_*i*_^S^ ≠ *I*_*j*_^S^, *k*_*i*_ ≠ *k*_*j*_. However, *k*_*i*_ may equal *k*_*j*_ due to the limited time window *T* and the noisy nature of the neural system. This will lead to the misidentification of the input values of *I*_*i*_^S^ and *I*_*j*_^S^. In other words, the input values *I*_*i*_^S^ and *I*_*j*_^S^ will be mixed or wrongly encoded in the output of neurons *i* and *j*. To avoid such confusion, the contrast of the firing rates of neurons *i* and *j* should be enhanced. We found in this paper that in an excitatory/inhibitory (E/I) network, the high-frequency synchronization of inhibitory interneurons can globally enhance the firing rate contrast of excitatory neurons, the model of which is described in Model of E/I Network. On the other hand, we hope that the neural system can transmit more information with less energy consumption, i.e., we hope neural systems have high information transmission efficiency. To characterize this efficiency, we propose a method based on the entropy theory, which is described in Methods for Quantifying Neuronal Information Transmission Efficiency.

### 2.1. Model of E/I Network

The network consists of one population of spiking excitatory neurons with size *N*_*e*_ and one population of spiking inhibitory neurons with size *N*_*i*_. Excitatory and inhibitory neurons receive synaptic inputs from both excitatory and inhibitory neurons. Neurons in the network connect to other neurons with a probability *ρ*. We define *W*^EI^ = (*w*_*i*,*j*_^EI^)_*N*_*i*_×*N*_*e*__ as the synaptic connection matrix from excitatory neurons to inhibitory neurons, where “E” in the superscript is the abbreviation of “excitatory neurons” and “I” is the abbreviation of “inhibitory neurons.” If there is a synapse from excitatory neuron *j* to inhibitory neuron *i*, *w*_*i*,*j*_^EI^ = *g*^EI^ with *g*^EI^ being the strength of synapse; otherwise, *w*_*i*,*j*_^EI^ = 0. The definitions for *W*^IE^ = (*w*_*i*,*j*_^IE^)_*N*_*e*_×*N*_*i*__, *w*_*i*,*j*_^IE^, *g*^IE^, *W*^EE^ = (*w*_*i*,*j*_^EE^)_*N*_*e*_×*N*_*e*__, *w*_*i*,*j*_^EE^, *g*^EE^, *W*^II^ = (*w*_*i*,*j*_^II^)_*N*_*i*_×*N*_*i*__, *w*_*i*,*j*_^II^, and *g*^II^ are similar to those of *W*^EI^ = (*w*_*i*,*j*_^EI^)_*N*_*i*_×*N*_*e*__, *w*_*i*,*j*_^EI^, and *g*^EI^.

The dynamics of the excitatory and inhibitory neurons are described as follows [29, 32].

(1) $$\begin{array}{c} \tau_{m}^{\text{E}}\frac{{dv}_{i}^{\text{E}}\left(t\right)}{dt}=-\left[v_{i}^{\text{E}}\left(t\right)-V_{\text{L}}\right]-R^{\text{E}}I_{i}^{\text{EE}}\left(t\right)-R^{\text{E}}I_{i}^{\text{IE}}\left(t\right)+R^{\text{E}}I_{i}^{\text{S}}\left(t\right), \end{array}$$

(2) $$\begin{array}{c} \tau_{m}^{\text{E}}\frac{{dv}_{i}^{\text{I}}\left(t\right)}{dt}=-\left[v_{i}^{\text{I}}\left(t\right)-V_{\text{L}}\right]-R^{\text{I}}I_{i}^{\text{EI}}\left(t\right)-R^{\text{I}}I_{i}^{\text{II}}\left(t\right)+R^{\text{I}}I_{i}^{B}\left(t\right). \end{array}$$

Here, *v*_*i*_^E^(*t*) and *v*_*i*_^I^(*t*) are the membrane potentials for the excitatory neurons and inhibitory neurons, respectively. When reaching the threshold potential *V*_th_, the neuron emits a spike, and the membrane potential returns to the rest potential *V*_rest_. *τ*_*m*_^E^ and *τ*_*m*_^I^ are the time constants of the membrane for the excitatory neurons and inhibitory neurons, respectively. *R*^E^ and *R*^I^ are the membrane resistances. *V*_L_ is the balance potential associated with the leak current. *I*_*i*_^EE^(*t*) is the summation of the synaptic current from all excitatory neurons to excitatory neuron *i*, and the definitions for *I*_*i*_^EI^(*t*), *I*_*i*_^IE^(*t*), and *I*_*i*_^II^(*t*) are similar to *I*_*i*_^EE^(*t*). They can be described as in

(3) $$\begin{array}{c} \begin{array}{c} I_{i}^{\text{EE}}\left(t\right)=\underset{j}{\sum }w_{i,j}^{\text{EE}}I_{i,j}^{\text{EE}}\left(t\right),\\ I_{i}^{\text{EI}}\left(t\right)=\underset{j}{\sum }w_{i,j}^{\text{EI}}I_{i,j}^{\text{EI}}\left(t\right),\\ I_{i}^{\text{IE}}\left(t\right)=\underset{j}{\sum }w_{i,j}^{\text{IE}}I_{i,j}^{\text{IE}}\left(t\right),\\ I_{i}^{\text{II}}\left(t\right)=\underset{j}{\sum }w_{i,j}^{\text{II}}I_{i,j}^{\text{II}}\left(t\right), \end{array} \end{array}$$

where *I*_*i*,*j*_^EE^(*t*) is the synaptic current resulting from all the spikes of neuron *j* [29]:

(4) $$\begin{array}{c} I_{i,j}^{\text{EE}}\left(t\right)=\underset{k}{\sum }\beta_{j,k}\left(t,\tau_{d}^{\text{E}},\tau_{r}^{\text{E}}\right)\left[v_{i}^{\text{E}}\left(t\right)-E_{\text{syn}}^{\text{E}}\right], \end{array}$$

(5) $$\begin{array}{c} \beta_{j,k}\left(t,\tau_{d}^{\text{E}},\tau_{r}^{\text{E}}\right)=\left\{\begin{array}{cc} 0, & t<t_{j,k}+d,\\ \frac{1}{\tau_{d}^{\text{E}}-\tau_{r}^{\text{E}}}\left(e^{-\left(t-t_{j,k}-d/\tau_{d}^{\text{E}}\right)}-e^{-\left(t-t_{j,k}-d/\tau_{r}^{\text{E}}\right)}\right), & t\geq t_{j,k}+d, \end{array}\right. \end{array}$$

where *t*_*j*,*k*_ is the firing time of the *k*-th spike of neuron *j*; *d* is the synaptic delay, *τ*_*d*_^E^ and *τ*_*r*_^E^ are the decay time constant and rise time constant of the excitatory synaptic current, respectively; and *E*_syn_^E^ is the reverse potential of the excitatory synapse.

Similar to the definition of *τ*_*d*_^E^, *τ*_*r*_^E^, and *E*_syn_^E^, we denote *τ*_*d*_^I^(*τ*_*r*_^I^) as the decay time constant (rise time constant) and *E*_syn_^I^ as the reverse potential of the inhibitory synapse. Correspondingly, the descriptions of *I*_*i*,*j*_^IE^(*t*), *I*_*i*,*j*_^EI^(*t*), and *I*_*i*,*j*_^II^(*t*) can be given similarly to that of *I*_*i*_^EE^(*t*).

*I*_*i*_^S^(*t*) is the stimulus presented to the excitatory neuron *i*. *I*_*i*_^S^(*t*) consists of two parts as follows:

(6) $$\begin{array}{c} I_{i}^{\text{S}}\left(t\right)=c_{i}^{\text{S}}\left(1+\varepsilon_{i}\left(t\right)\right), \end{array}$$

where *c*_*i*_^S^ is the strength of the stimulus. It is a constant, but each neuron has a different strength, i.e., *c*_*i*_^S^ ≠ *c*_*j*_^S^. *c*_*i*_^S^ is drawn from a normal distribution with standard deviation *δ*^S^ and mean *m*^S^. *ε*_*i*_(*t*) is white noise, which obeys a normal distribution with standard deviation *σ*_N_^S^ and a mean of zero. The parameter *δ*^S^ controls the contrast of the strength of the stimuli. Larger *δ*^S^ means higher contrast of the strength of the stimuli.

Similar to *I*_*i*_^S^(*t*), the background input to the inhibitory neurons *I*_*i*_^B^(*t*) is defined; thereby, we have parameters *δ*^B^, *m*^B^, and *δ*_N_^B^ for *I*_*i*_^B^. The excitatory neurons may have background input as inhibitory neurons, and here, we assume that *I*_*i*_^S^(*t*) includes this background input.

### 2.2. Methods for Quantifying Neuronal Information Transmission Efficiency

To simplify the descriptions of the method, assume that the inputs of the neurons are images, which are denoted by *X* = {*x*_*i*_, *i* = 1, 2, ⋯, *N*_*e*_}, where *X* represents the image presented to the neurons, *N*_*e*_ is the number of pixels in an image, and *x*_*i*_ is the gray value of the *i-*th pixel in the image. The output of the neurons represents the output image with the number of firings of neuron *i* during time window *T*. *k*_*i*_ stands for the gray value of the corresponding pixel. The images are presented to the neurons by letting *I*_*j*_^S^ = *x*_*j*_. Suppose that the input *x*_*i*_ is discrete (continuous values of the gray value can be discretized following the degree of the resolution in the neural systems), i.e., *x*_*i*_ ∈ {0, Δ*x*, 2Δ*x*, ⋯, *M*Δ*x*} where Δ*x* is the resolution of the discretization. Let us denote *s*_*j*_ = *j*Δ*x*. Let *L*_*g*_(*i*, *j*) = 1 if the gray value of the *i*-th pixel is *j*Δ*x*; otherwise, *L*_*g*_(*i*, *j*) = 0. Let *p*(*s*_*j*_) = ∑_*i*=1_^*N*_*e*_^*L*_*g*_(*i*, *j*)/*N*_*e*_ be the “probability” (proportion) of the pixels whose gray value is *j*Δ*x*. Then, the entropy of the input image (stimulus) can be calculated as *S*_S_ = −∑_*j*=1_^*M*^*p*(*s*_*j*_)log(*p*(*s*_*j*_)). Let *p*(*k*) be the “probability” (proportion) of neurons whose spiking number is *k*. Then, the full entropy of the output image is defined as *S*_full_ = −∑_*k*=0_^∞^*p*(*k*) log(*p*(*k*) ). If for any pair of neurons with *I*_*i*_^S^ ≠ *I*_*j*_^S^, *k*_*i*_ ≠ *k*_*j*_, i.e., if the different inputs of any two neurons can be distinguished by their outputs, then the mutual information, *S*_I_, will be equal to the entropy of the input image, *S*_I_ = *S*_S_. This means that the information is transmitted from the input side of the neural system to the output side of the neural system without any loss. However, as we discussed in the first paragraph of Model and Methods, *k*_*i*_ may be equal to *k*_*j*_ when *I*_*i*_^S^ ≠ *I*_*j*_^S^, which results in *S*_I_ < *S*_S_, implying that some information is lost due to the finite time window and noisy nature of the neuronal channels. The corresponding noise entropy, which is denoted by *S*_noise_, can be calculated as *S*_noise_ = −∑_*j*=1_^*M*^∑_*k*=0_^∞^*p*(*s*_*j*_)*p*(*k* | *s*_*j*_)log(*p*(*k* | *s*_*j*_)) according to the entropy theory [29]. Here, *p*(*k* | *s*_*j*_) is defined as *p*(*k* | *s*_*j*_) = *C*_1_/*C*_2_, where *C*_1_ is the count of neurons whose spike number is *k* and input is *s*_*j*_, and *C*_2_ is the count of neurons whose input is *s*_*j*_. The mutual information is the full entropy subtracted by noise entropy, *S*_I_ = *S*_full_ − *S*_noise_.

Energy consumption [33, 34] should also be considered when neural systems encode information with firing rates [35–37]. It is desired that neural systems carry more information with less energy consumption. Metabolic energy is usually considered when encoding efficiency is investigated, which is quantified by the number of spikes employed to encode the information. Then, the information efficiency is calculated as *I*_E_ = *S*_I_/*F*_S_, where *F*_S_ is the average number of spikes of neurons.

## 3. Simulation Results

### 3.1. Firing Patterns in a Fully Connected Network

We first use a relatively simple E/I network to focus our presentation on our idea of global contrast enhancement resulting from the high-frequency synchronization, where neurons are fully connected and the background inputs to the inhibitory neurons are homogeneous. The parameter values are set similar to Ref. [29], which are set as follows unless otherwise stated: *ρ* = 1, *N*_*e*_ = 450, *N*_*i*_ = 150, *g*^EI^ = *g*^EE^ = 1.2/(*N*_*e*_ + *N*_*i*_), *g*^IE^ = *g*^II^ = 7/(*N*_*e*_ + *N*_*i*_), *τ*_*m*_^E^ = 50 ms, *τ*_*m*_^I^ = 10 ms, *R*^E^ = *R*^I^ = 1 M*Ω*, *V*_L_ = −65 mV, *τ*_*d*_^E^ = *τ*_*d*_^I^ = 6 ms, *τ*_*r*_^E^ = *τ*_*r*_^I^ = 0.1 ms, *E*_syn_^E^ = 0 mV, *E*_syn_^I^ = −85 mV,*d* = 0.2 ms,*δ*^S^ = 0.5*m*^S^ = 4, *δ*_N_^S^ = 0.5,*δ*^B^ = 0, *m*^B^ = 2.4, *σ*_N_^B^ = 0, and *T* = 300 ms. It should be notified that the numbers of excitatory neurons and inhibitory neurons can be changed (the ratio of the two types of neurons falls in a range around 3 : 1~4 : 1 according to Ref. [38], and we choose 3 : 1 in this paper). If the ratio of excitatory neurons and inhibitory neurons is changed, the excitatory and inhibitory synaptic strengths should also be suitably changed to maintain the balance of excitation and inhibition in the network so that the network still oscillates synchronously.

**Figure 2:**
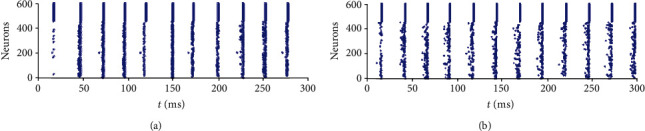
High-frequency oscillation in E/I networks with (a) no noisy input to the excitatory neurons, *σ*_*N*_^*S*^ = 0, and (b) noisy input to the excitatory neurons, *σ*_*N*_^*S*^ = 0.5.

Figure 2 shows the raster plots of the firing of neurons in the E/I network, where neurons labeled from 1 to 450 are excitatory neurons and neurons labeled from 451 to 600 are inhibitory neurons. Inhibitory neurons fire synchronously and act as the globally inhibitory signal to all the neurons in the E/I network. In each cycle of synchronization, a fraction of excitatory neurons fires, and it is the firings of these neurons that drive the inhibitory neurons to fire in this cycle of synchronization. Neurons with larger input will fire more frequently than those with smaller input, i.e., neurons with larger input will fire in more cycles of synchronization. Thus, the input values are encoded in the firing rates (the number of spikes) of these excitatory neurons.

### 3.2. Contrast Enhancement in a Fully Connected Network

**Figure 3:**
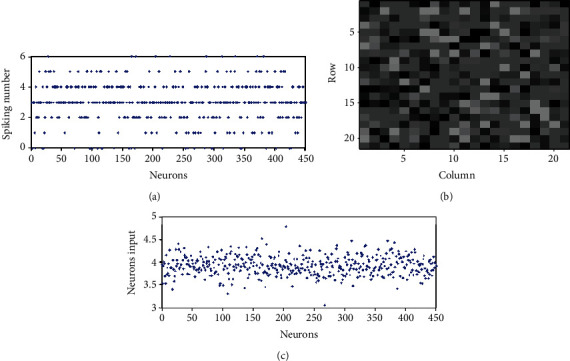
Firing rates of all excitatory neurons in E/I networks with no noise, *σ*_*N*_^*S*^ = 0: (a) spiking numbers of all excitatory neurons within 300 ms; (b) visualization of the firing rates of neurons 1-400 with gray values located at (*i*, *j*) representing the firing rate of neurons 20∗(*i* − 1) + *j*; (c) inputs of neurons 1-450.

**Figure 4:**
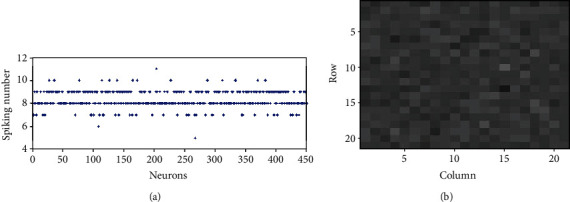
Firing rates of all excitatory neurons of a population of isolated excitatory neurons with no noise, *σ*_*N*_^*S*^ = 0: (a) spiking numbers of all excitatory neurons within 300 ms; (b) visualization of the firing rates of neurons 1-400.

We first investigate the firing rate contrast in the fully connected network when randomness is not included in the stimulus to the excitatory neurons, i.e., *σ*_N_^S^ = 0.  Figure 3(a) shows the number of spikes of all neurons when *T* = 300 ms in such cases. It is very interesting and unexpected to see that the number of spikes of all the neurons scattered between 0 and 6, as the inputs of all the neurons are clustered around 4 (see Figure 3(c)). For comparison, Figure 4(a) shows the number of spikes of all the neurons when neurons do not connect with each other, i.e., each neuron is isolated and only receives the external input *I*_*i*_^S^(*t*) in Equation (1), and the synaptic inputs *I*_*i*_^EE^(*t*) and *I*_*i*_^IE^(*t*) are both 0. We can see from Figure 4(a) that the number of spikes of isolated neurons is closely around 8 and 9. Stated in other words, the contrast of the number of spikes of excitatory neurons in the E/I network is enhanced compared with that of the isolated neurons. If we measure the number of spikes when *T* is large enough, we can get the firing rate of each neuron, *r*_*i*_ ≈ *k*_*i*_/*T*. Figures 3(b) and 4(b) visualize the firing rates of 400 excitatory neurons in the E/I network and a population of isolated neurons, respectively, where the gray value of the small square at the position (*i*,*j*) represents the firing rate of the neuron with index 20∗(*i* − 1) + *j*. It shows clearly that the gray contrast of Figure 3(b) is much larger than that of Figure 4(b), implying that the firing rate contrast of excitatory neurons in the E/I network is much larger than those in the population of isolated neurons.

**Figure 5:**
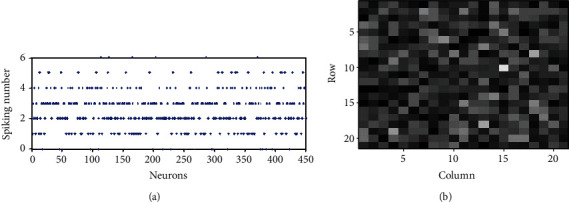
Firing rates of all excitatory neurons in E/I networks with noisy input to excitatory neurons, *σ*_*N*_^*S*^ = 0.5: (a) spiking numbers of all excitatory neurons within 300 ms; (b) visualization of the firing rates of neurons 1-400.

**Figure 6:**
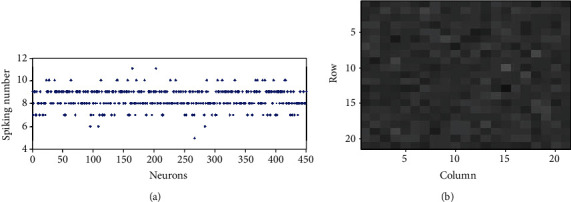
Firing rates of all excitatory neurons of a population of isolated excitatory neurons with noisy input to excitatory neurons, *σ*_*N*_^*S*^ = 0.5: (a) spiking numbers of all excitatory neurons within 300 ms; (b) visualization of the firing rates of neurons 1-400.

**Figure 7:**
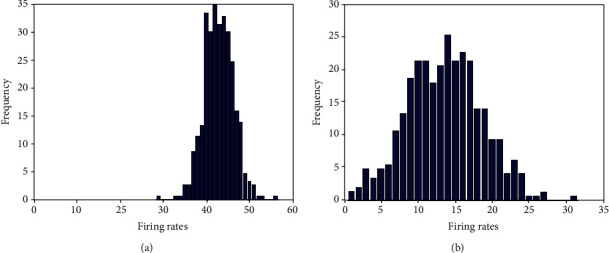
Histograms of the firing rates of all excitatory neurons in (a) a population of isolated excitatory neurons with noisy input and (b) E/I networks with noisy input.

The enhancement of the contrast of the firing rates of excitatory neurons in E/I networks when randomness is included is also confirmed in Figures 5 and 6, where the spiking numbers of neurons within time window *T* and visualization of the firing rates are shown. Randomness is introduced to the network by setting *σ*_N_^S^ = 0.5. Figures 7(a) and 7(b) further show the histograms of the firing rates of the excitatory neurons in the E/I network and in the population of isolated neurons, respectively. The histogram of Figure 7(b) is more homogeneous than that of Figure 7(a), also confirming that the firing rate contrast of excitatory neurons in the E/I network is much larger than that in the population of isolated neurons.

To get a clearer watch of this contrast enhancement, we arbitrarily choose several neurons between Figures 3(a) and 4(a), say neurons 6 to 15, and display the firing rates of the ten neurons in Table 1. We can see that the number of spikes of the isolated neurons only has three values, 7, 8, or 9. Some neurons have an identical number of spikes though their inputs are somewhat different. Namely, the number of spikes of some neurons overlaps, resulting in a failure of the encoding of the inputs of these neurons. For example, both neuron 6 and neuron 15 have 8 spikes though they have different inputs. This is because the contrast of the firing rates of the two neurons (neuron 6 and neuron 15 have firing rates of 25.3 and 27.3, respectively) is so small that the number of the spikes within a small time window *T* cannot be distinguished. On the other hand, as the contrast of the firing rates of the excitatory neurons in the E/I network is enhanced, the problem of this overlapping is alleviated. The number of spikes of these neurons in the E/I network has more individual values (0, 1, 2, 3, 4, or 5) than those (7, 8, or 9) in a population of isolated neurons (notice that as the synaptic inhibition in the E/I network is stronger than excitation, the number of spikes fired by neurons in the E/I network is less than those in the population of isolated neurons). The numbers of spikes of neuron 6 and neuron 15 are no longer identical (they have 1 and 3 spikes, respectively); this is because they have a relatively large contrast of the firing rates (they have firing rates of 5.3 and 12.7, respectively). Therefore, thanks to the enhancement of the contrast of the firing rates, the output overlapping problem is somewhat relieved. Correspondingly, it is found that the information transmission efficiency is much higher in the E/I network (it is 0.54 in the E/I network of Figure 3(a)) than that in the population of isolated neurons (it is 0.13 in the population of isolated neurons of Figure 4(a)).

There is another more important problem, the noise disturbance problem, for the information transmission caused by the small contrast of firing rates in a population of isolated neurons. Noise will disturb the encoding of the input information. If the difference of the firing rates of the two neurons is too small, the noise will make the two neurons fire the same number of spikes. Similarly, as in Table 1, we choose 10 neurons from Figures 5(a) and 6(a) and display their firing rates in Table 2. It can be seen that the firing rate of neuron 23 (26.5) is a little larger than that of neuron 21 (24.0), but the noise makes neuron 23 emit the same number of spikes (8 spikes) as neuron 21 within the small time window *T*. As a result, it fails for the identification of the two inputs. This noise disturbance problem can also be alleviated in an E/I network. As the contrast of the firing rates of neuron 23 and neuron 21 is enhanced in an E/I network (they have firing rates of 6.7 and 2, respectively), noise cannot make neuron 23 fire the same spikes as neuron 21 any longer (neuron 23 and neuron 21 fire 3 and 1 spikes, respectively); thereby, we can distinguish the strength of the inputs of the two neurons by their outputs correctly. Therefore, due to the alleviation of the output overlapping problem and noise disturbance problem, the information efficiency in the E/I network (it is 0.39 in the E/I network of Figure 5(a)) is greatly improved compared to that in a population of isolated neurons (it is 0.098 in the population of isolated neurons of Figure 6(a)).

### 3.3. Contrast Enhancement in a Randomly Connected Network

**Figure 8:**
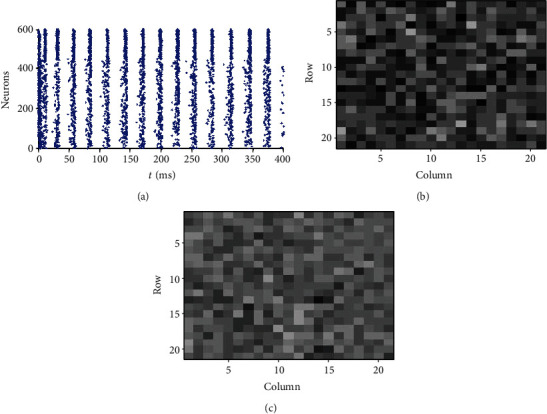
Firing rate contrast enhancement in a randomly connected E/I network: (a) raster plot of the firing of all neurons; (b) visualization of the firing rates of neurons 1-400 in the E/I network; (c) visualization of the firing rates of neurons 1-400 in a population of isolated neurons. *ρ* = 0.7 , *δ*^*B*^ = 0.1, *σ*_*N*_^*B*^ = 0.5, *τ*_*d*_^*E*^ = 20 ms, *τ*_*d*_^*I*^ = 5 ms, *g*^EI^ = *g*^EI^ = 3/(*N*_*e*_ + *N*_*i*_), *g*^IE^ = *g*^II^ = 20/(*N*_*e*_ + *N*_*i*_), *τ*_*m*_^*I*^ = 25 ms, *E*_syn_^*I*^ = −75 mV, *d* = 3.5 ms, and *m*^*S*^ = 3. The other parameters are set the same as those in Figure 5.

It is worthwhile noting that although the contrast enhancement is explored in the relatively simple E/I network, the results obtained in this paper are also reserved in more realistic E/I networks.  Figure 8 shows the firing rate distribution and information transmission efficiency in a randomly connected network with the connection probability *ρ* = 0.7 among all the neurons. The input to each inhibitory neuron is different one by one by setting *δ*^B^ = 0.1, *σ*_N_^B^ = 0.5. The decay constants of excitatory synapses and inhibitory synapses are set differently as *τ*_*d*_^E^ = 20 ms, *τ*_*d*_^I^ = 5 ms.  Figure 8(a) shows that inhibitory neurons fire synchronously, and a fraction of excitatory neurons fires at each cycle like the relatively simple case in Figure 2.  Figure 8(b) visualizes the number of spikes of neurons 1~400 similar to Figure 5(b), which reveals the contrast enhancement of the firing rates of the excitatory neurons in the E/I network, compared with Figure 8(c). The information transmission efficiency is also calculated. The information transmission efficiency in the E/I network is 2.3 times of that in a population of isolated neurons.

### 3.4. Information Transmission Efficiency

**Figure 9:**
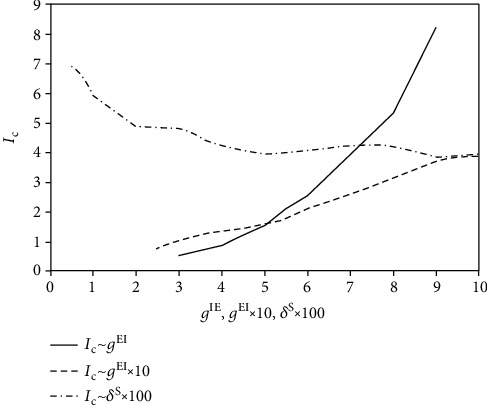
Dependency of information encoding efficiency on (a) strength of the synapse from inhibitory neurons to excitatory neurons, (b) strength of the synapse from excitatory neurons to inhibitory neurons, and (c) standard deviation of the stimuli.

Further, we explored the dependency of the information transmission efficiency on the parameter values. Firstly, we checked the effect of two important parameters of the network on the efficiency of information encoding of the excitatory neurons, i.e., the strength of the inhibition to the excitatory neurons and the strength of the synaptic currents from the excitatory neurons to the inhibitory neurons. To compare the information transmission efficiency of the E/I network with that of a population of isolated neurons, we define an information transmission efficiency quantity *I*_C_ = *I*_E_/*I*_S_, where *I*_E_ is the information transmission efficiency of the E/I network and *I*_S_ is the information transmission efficiency of the corresponding population of isolated neurons.  Figure 9 shows that *I*_C_ declines when the strength of the inhibition to the excitatory neurons decreases (see the solid line). *I*_C_ is even smaller than 1 when the strength of inhibition is too weak, implying that the information transmission efficiency of an E/I network with no inhibition to the excitatory neurons is worse than a population of isolated neurons. This is because without inhibition, excitatory synaptic currents, which are approximately the same to each neuron, may even homogenize the contrast of the inputs. Therefore, inhibition is important for high information transmission efficiency.  Figure 9 also shows that *I*_C_ declines when the strength of the synaptic current from the excitatory to the inhibitory neurons decreases (see the dashed line). This means that the inputs from the excitatory neurons to the inhibitory neurons are also necessary for high efficiency. Hence, synaptic inhibition and excitation work together to enhance the contrast, which we will explain in the next section.

## 4. Mechanism of the Global Contrast Enhancement

**Figure 10:**
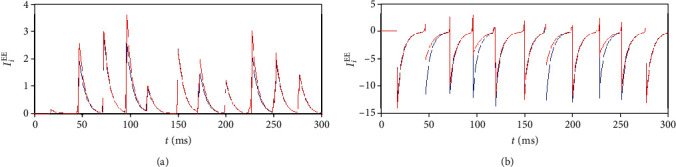
Comparisons of the synaptic currents received by a neuron with a higher firing rate (red) and a neuron with a slower firing rate (blue): (a) total synaptic currents from excitatory neurons; (b) total synaptic currents from inhibitory neurons.

One may wonder how the global contrast enhancement happens as neurons receive the same afferent synaptic spikes from the network (notice that each neuron connects to all the other neurons in the network)? We found by close observation of the synaptic currents of two typical neurons (the firing rate of one of them is higher than the other) that the synaptic currents of the two neurons are different although they receive the same afferent synaptic spikes. It is this difference between the synaptic currents that results in the contrast enhancement. The synaptic current of a neuron is divided into two parts. One part is the excitatory current due to spikes from the excitatory neurons (see Figure 10(a)), and the other part is the inhibitory current due to spikes from the inhibitory neurons (see Figure 10(b)). Figure 10(a) shows that the excitatory current of the neuron with a higher firing rate receives a larger excitatory synaptic current, and the neuron with a lower firing rate receives a smaller current. On the other hand, Figure 10(b) shows that the neuron with a higher firing rate receives a smaller negative synaptic current while the neuron with a lower firing rate receives a larger negative synaptic current. Combining the two elements together, we can conclude that the neuron with the higher firing rate receives more synaptic excitation and less synaptic inhibition, but the neuron with the lower firing rate receives less excitation and more inhibition. Consequently, the neurons with higher firing rates in the network will fire with even higher rates, but neurons with lower firing rates will fire with even lower rates. This explains the firing rate contrast enhancement we obtained in Simulation Results in this paper.

The next interesting question is why synaptic currents are different for the two neurons although the afferent spikes for them are the same? This can be explained by the voltage-dependent properties of the synaptic currents [39]. Let us consider the synaptic currents received by a neuron after it fires at a certain cycle of synchronization. For example, neuron 1 fires at the time *t*_*k*_; therefore, the membrane potential of neuron 1 resumes to the reset potential (-65 mV in the model) immediately after its firing. As synchronization of the firing of the neurons also occurs at roughly *t*_*k*_, the inhibitory currents induced by the synchronized spikes from the inhibitory neurons will be small due to the low membrane potential of neuron 1 according to Equation (4) (notice that for inhibitory synapse, *E*_syn_^E^ should be replaced by *E*_syn_^I^). On the other hand, neuron 2, which does not fire at *t*_*k*_, will receive a larger inhibitory current due to the higher membrane potential compared to neuron 1. Namely, a neuron firing at a cycle of synchronization will receive a smaller negative synaptic current than those who do not fire, implying that neurons with higher firing rates will receive smaller negative inhibitory currents.

In short words, it is the voltage-dependent property of the synaptic current that causes high firing rate neurons to receive large excitatory synaptic currents and small inhibitory synaptic currents, thereby firing with even higher firing rates. Similarly, low firing rate neurons receive small excitatory synaptic currents and large inhibitory synaptic currents, thereby firing with even lower firing rates. This may be the mechanism that explains the firing rate contrast enhancement exhibited in the synchronized E/I network in this paper.

## 5. Conclusions and Discussions

Synchronization with high frequency in excitatory/inhibitory networks has been found in many real neural systems, which is pertinent to some high-frequency components of neuronal oscillation, for example, gamma oscillation. Some possible functional roles of this high-frequency synchronization have been hypothesized and have been tested in theoretical models [12–14]. Contrast enhancement is a general feature of information processing in neural systems, especially in sensory pathways [23]. We found that high-frequency synchronization in the E/I network can enhance the contrast of the firing rates of excitatory neurons globally. Lateral inhibition is widely believed to be able to enhance the contrast of the firing rates of neighboring neurons [24, 25]. It is supposed that information is encoded in excitatory neurons, and neurons only connect to neighboring neurons. The excitatory neurons activate neighboring inhibitory neurons, which in turn inhibit neighboring excitatory neurons. Thereby, excitatory neurons with a higher firing rate may inhibit neighboring neurons more, therefore causing the following effect: for two neighboring excitatory neurons, the one with a lower firing rate receives more inhibition while the other with a higher firing rate receives less inhibition. This reciprocal inhibition of neighboring neurons enhances their firing rate contrast. Unlike lateral inhibition, where neurons only connect to neighboring ones resulting in local firing rate contrast enhancement, each neuron connects to all the other neurons in the network in our model (or connects to all neurons with a constant probability as in the model), resulting in global contrast enhancement. Namely, the inhibition caused by the synchronized inhibitory neurons enhances the firing rate contrast of all excitatory neurons in the network in our model, whereas lateral inhibition only enhances the firing rate contrast of the corresponding two neighboring neurons.

The firing rate contrast of all excitatory neurons in the network can be characterized by the theory of image entropy. If we view the firing rate of each neuron as the gray value of a pixel in an image, then the firing rates of all excitatory neurons can project a gray-valued image. Since an image with high image entropy has high image contrast, we can use the theory of image entropy to characterize the firing rate contrast of all excitatory neurons. Correspondingly, the information transmission efficiency is relevant to the entropy of the inputs and the outputs (firing rates) of the neurons. We found that the global contrast enhancement in this paper can alleviate two problems. One is the output overlap problem that the spiking numbers of neurons with a low firing rate contrast will be identical. The other is the noise disturbance problem that noise will make neurons with different inputs fire the same number of spikes. Thus, the contrast enhancement in the E/I networks can improve the information transmission efficiency, namely, increase the mutual information on the one hand and decrease the energy consumption on the other hand. Another important thing for real neural systems is that they need to distinguish signals with high similarities. In our model, signals with high similarities mean that the parameter value *δ*^S^ is small (if *δ*^S^ is small, the input contrast of all excitatory neurons is small). We can see from Figure 9 (see the dot dashed line) that the information transmission efficiency increases with the decrease of this parameter, implying that the information transmission efficiency is enhanced more by the E/I network if the input contrast is lower. This has a strong biological implication because the neural systems need to enhance the contrast of sensory input more for clear identification of the signals when the sensory input contrast is smaller.

The mechanism of contrast enhancement is also analyzed in the paper. The global contrast enhancement results from the interaction of the excitatory neurons and inhibitory neurons. Inhibitory neurons fire synchronously at each cycle, while only a fraction of excitatory neurons fires at each cycle. It is the firing of these excitatory neurons that drives the synchronized firing of the inhibitory neurons at each cycle of the synchronization, and the firing inhibitory neurons inhibit all neurons in the network. Due to the voltage-dependent properties of the synaptic currents, the inhibitory synaptic currents to the fired excitatory neurons are less than those of the nonfiring excitatory neurons. Thus, neurons that fire more frequently may receive less inhibition, resulting in the enhancement of the contrast of the firing rates.

Our work implies a possible functional role in information transmission of high-frequency synchronization in neuronal systems and also might be heuristic for modelling of an artificial neuron model [40] and neural networks [41], which will be explored in our future work.

## Figures and Tables

**Table 1:** Spiking numbers of 10 isolated neurons and 10 excitatory neurons in E/I networks (see Figures 3(a) and 4(a)) with no noise.

Neuron index	6	7	8	9	10	11	12	13	14	15
Strength of the stimuli	3.69	3.53	4.16	3.73	3.87	3.75	3.96	3.70	4.26	3.88
Spiking numbers of isolated neurons	8	7	9	8	8	8	8	8	9	8
Spiking numbers of neurons in E/I network	1	0	4	2	3	2	3	2	5	3

**Table 2:** Spiking numbers of 10 isolated neurons and 10 excitatory neurons in E/I networks (see Figures 5(a) and 6(a)) with noise.

Neuron index	21	22	23	24	25	26	27	28	29	30
Strength of the stimuli	3.58	3.69	3.80	4.27	3.91	4.03	3.97	4.40	4.28	3.84
Spiking numbers of isolated neurons	8	7	8	10	9	9	9	10	10	8
Spiking numbers of neurons in E/I network	1	2	3	4	3	4	3	5	5	3

## Data Availability

The data is available upon request.
